# Identification of histone methyltransferase NSD2 as an important oncogenic gene in colorectal cancer

**DOI:** 10.1038/s41419-021-04267-6

**Published:** 2021-10-20

**Authors:** Li-hao Zhao, Quan Li, Zhi-Jun Huang, Mi-Xue Sun, Jing-jing Lu, Xiao-hua Zhang, Gang Li, Fang Wu

**Affiliations:** 1 Department of Radiation Oncology, The First Affiliated Hospital of Wenzhou Medical University, Wenzhou, Zhejiang China; 2 Center of Stomatology, The Second Affiliated Hospital of Soochow University, Suzhou, China; 3 Department of Surgery, Yancheng First People’s Hospital, Yancheng, China; 4 Department of Radiotherapy and Oncology, Affiliated Kunshan Hospital of Jiangsu University, Kunshan, China; 5 Department of Surgical Oncology, The First Affiliated Hospital of Wenzhou Medical University, Wenzhou, Zhejiang China; 6 Department of Gastroenterology, The First Affiliated Hospital of Wenzhou Medical University, Wenzhou, China

**Keywords:** Colon cancer, Oncogenes

## Abstract

Colorectal cancer (CRC) is the second common cause of cancer-related human mortalities. Dysregulation of histone 3 (H3) methylation could lead to transcriptional activation of multiple oncogenes, which is closely associated with CRC tumorigenesis and progression. Nuclear receptor-binding SET Domain protein 2 (NSD2) is a key histone methyltransferase catalyzing histone H3 lysine 36 dimethylation (H3K36me2). Its expression, the potential functions, and molecular mechanisms in CRC are studied here. Gene Expression Profiling Interactive Analysis (GEPIA) bioinformatics results showed that the *NSD2* mRNA expression is elevated in both colon cancers and rectal cancers. Furthermore, *NSD2* mRNA and protein expression levels in local colon cancer tissues are significantly higher than those in matched surrounding normal tissues. In primary human colon cancer cells and established CRC cell lines, shRNA-induced silencing or CRISPR/Cas9-induced knockout of NSD2 inhibited cell viability, proliferation, cell cycle progression, migration, and invasion. Furthermore, NSD2 shRNA or knockout induced mitochondrial depolarization, DNA damage, and apoptosis in the primary and established CRC cells. Contrarily, ectopic NSD2 overexpression in primary colon cancer cells further enhanced cell proliferation, migration, and invasion. H3K36me2, expressions of multiple oncogenes (*ADAM9*, *EGFR*, *Sox2*, *Bcl-2*, *SYK*, and *MET*) and Akt activation were significantly decreased after NSD2 silencing or knockout in primary colon cancer cells. Their levels were however increased after ectopic NSD2 overexpression. A catalytic inactive NSD2 (Y1179A) also inhibited H3K36me2, multiple oncogenes expression, and Akt activation, as well as cell proliferation and migration in primary colon cancer cells. In vivo, intratumoral injection of adeno-associated virus (AAV)-packed NSD2 shRNA largely inhibited primary colon cancer cell xenograft growth in nude mice. Together, NSD2 exerted oncogenic functions in CRC and could be a promising therapeutic target.

## Introduction

Colorectal cancer (CRC) is the second most common cause of cancer mortalities around the world [1, 2]. In the United States alone, it is estimated that over 147,000 individuals will be diagnosed with CRC in 2020. Over 53,000 patients will die from this devastating disease, including about 18,000 cases and 3500 deaths in younger individuals (aged 50 years and under) [3]. In the past decade, the death rate of CRC declined 3% annually in the older individuals (over 65 years), but increased by 1.3% annually in younger CRC patients [3]. For the advanced and recurrent CRC patients, the prognosis and 5-year overall survival (OS) are however far from satisfactory [4, 5].

CRC molecular heterogeneity is one important reason of failure to specific molecularly targeted agents [5, 6]. Therefore, it is urgent to further explore the underlying mechanisms responsible for tumorigenesis and progression of CRC [5, 6]. It is also the research focus of our group [7, 8]. Dysregulation and/or aberrant expression of histone methyltransferases or demethylases will cause perturbations in histone methylation status, which is a characteristic marker of human cancer [9, 10]. Nuclear receptor-binding SET domain protein 2 (NSD2) is a primary member of SET domain-containing methyltransferases. NSD2 catalyzes histone 3 lysine 36 dimethylation (H3K36me2) [11], required for the transcriptional activation of certain genes (including multiple oncogenes) [12–17].

Studies have reported that NSD2 functions as an important oncogene and is overexpressed in several solid tumors [12–17]. NSD2 positively regulates cell proliferation, migration, invasion, and epithelial-mesenchymal transformation (EMT) [12–17]. Contrarily, NSD2 silencing or inhibition efficiently inhibited the tumorigenesis and progression of different cancers [12–17]. Aytes et al. [18] have shown that NSD2 expression is upregulated in prostate cancer, which is associated with poor prognosis. NSD2 silencing by specific short hairpin RNA (shRNA) inhibited prostate cancer cell growth, proliferation, and migration in vitro, and abrogated prostate cancer metastasis in vivo [18]. Han et al. [13] showed that NSD2 promoted clear cell renal cell carcinoma (ccRCC) cell growth and progression via activating the Akt-Erk cascade. NSD2 overexpression is detected in human ccRCC, correlated with poor OS [13]. NSD2 is also a putative cofactor of androgen receptor [19], important for advanced prostate cancer progression [20–22]. NSD2 overexpression is detected in close to 80% of prostate cancer, which is correlated with prostate-specific antigen (PSA) progression and poor OS [12]. Moreover, NSD2 inhibition suppressed cervical cancer tumorigenesis and metastasis [23], and reduced expressions of transforming growth factor-β1 (TGF-β1) and its receptor TGF-βRI [23].

In addition, NSD2 could also collaborate with oncogenic RAS in the lung cancer cells, to enhance epigenetic activation [24]. Zhang et al. [25] showed that NSD2 mediated phosphatase and tensin homolog (PTEN) dimethylation to dictate PTEN recruitment to the DNA-damage sites, which is essential for the efficient repair of DNA double-strand breaks [25]. NSD2 silencing sensitized cancer cells to combinatorial treatment with a phosphoinositide 3-kinase (PI3K) inhibitor and DNA-damaging agent [25]. Therefore, these studies have proposed the oncogenic role of NSD2. The expression and potential oncogenic functions of the histone methyltransferase in CRC were examined here.

## Materials and methods

### Materials, reagents, and antibodies

Puromycin, neomycin, and polybrene were provided by Sigma-Aldrich (St. Louis, MO). Fetal bovine serum (FBS), Dulbecco’s modified Eagle’s medium (DMEM), RPMI, and other cell culture reagents were obtained from Gibco BRL (Grand Island, NY). Antibodies for NSD2 (#65127), Histone H3 (#9927), H3K36me2 (#2901), cleaved caspase-3 (#9664), cleaved-poly (ADP-ribose) polymerase (PARP) (#5625), cleaved caspase-9 (#20750), β-actin (#4970), and Tubulin (#2125), as well as phosphorylated-Akt Ser-473 (#9271) and Akt1/2 (#9272), were obtained from Cell Signaling Tech (Shanghai, China).

### Cell culture

HCT-116 and HT-29 cell lines were provided by Dr. Lu at Nanjing Medical University [26]. Cells were cultured in DMEM medium plus 10% FBS. The primary human colon cancer cells (derived from three primary colon cancer patients, namely “pri-Can-1/-2/-3”) and the primary human colon epithelial cells (“pri-Epi,” from one independent donor) were from Dr. Lu as well [26–28]. The protocols of culturing primary human cell were described previously [29]. Using of human cells was according to the principles of Declaration of Helsinki, with approval from the Ethics Committee of Wenzhou Medical University. The written informed consent was obtained from each participant who were providing tissues.

### Human tissues

The colon cancer tissues and matched surrounding normal colon epithelial tissues, from 20 independent primary colon cancer patients (male, stage II–III, 45–62 years old), were reported in our previous studies [7, 8]). Tissue lysates were stored in liquid nitrogen and were subjected to biomedical analyses. Written informed consent was obtained from each patient. The protocols of using human tissues were according to the principles of Declaration of Helsinki and were approved by the Ethics Committee of Wenzhou Medical University.

### qRT-PCR analyses

Total RNA from CRC cells with the applied genetic modifications or the fresh tissue specimens was extracted by using the TRIzol reagent (Invitrogen; Thermo Fisher Scientific, Shanghai, China). The synthesis of complementary DNA (cDNA) was performed by using a PrimeScript RT Reagent kit (Takara Bio, Inc.). Quantitative reverse-transcription PCR (qRT-PCR) was performed under the ABI 7900 Fast Real-Time PCR system (Applied Bioscience; Thermo Fisher Scientific) using the Power SYBR Green PCR Master mix kit (Applied Biosystems). The relative mRNA expression was examined using a standard 2^−ΔΔCt^ method after normalizing to *glyceraldehyde-3-phosphate dehydrogenase*. The primers utilized in this study were synthesized from Genechem (Shanghai, China).

### Western blotting

Protein lysates (25 µg per sample in each lane) were loaded in denaturing 10–12.5% SDS-polyacrylamide gel electrophoresis gels and transferred to polyvinylidene dichloride blots (Millipore, Shanghai, China). Membranes were incubated with 10% non-fat milk for 1 h at room temperature, washed with phosphate-buffered saline containing Tween 20 (PBST) and then incubated with the indicated primary antibodies overnight. After washing, the blots were incubated with corresponding secondary antibodies for 2 h at room temperature. A Pierce ECL Plus kit (Shanghai, China) was utilized to detect protein signals under a chemiluminescence system (Amersham).

### NSD2 shRNA

A set of two different shRNAs targeting non-overlapping sequences of NSD2 (sequences were listed in a previous study [18]) were individually annealed into a GV369 vector (Genechem, Shanghai, China). The construct was then transfected to HEK-293 cells together with lentiviral Helper 1.0 and Helper 2.0 plasmids (Genechem, Shanghai, China) via Lipofectamine 3000 (Thermo Fisher Scientific, Shanghai, China) for 48 h. Lentiviral particles, at multiplicity of infection (MOI) of 10, were then added to CRC cells (cultured in polybrene-containing complete medium) for 24 h. Stable cells were selected by puromycin (2.5 μg/mL) for four passages. NSD2 silencing in the stable cells was verified by qRT-PCR and western blotting assays. Control cells were infected with the scramble control shRNA lentiviral particles (Santa Cruz Biotech, Santa Cruz, CA). For in vivo studies, an adeno-associated virus (AAV) construct (AAV9, Genechem, Shanghai, China) was utilized and the NSD2 shRNA sequence was inserted into the construct. The shRNA AAV was generated by transfection of the construct to HEK-293 cells along with the AAV package plasmids (Genechem). The virus was filtered, enriched, and injected to tumor xenografts in mice.

### CRISPR/Cas9-mediated NSD2 knockout

CRC cells were first infected with LV-Cas9 lentivirus (Genechem, Shanghai, China) at an MOI of 8 for 48 h and selected by puromycin (5 µg/mL) for three passages. LV-Cas9-expressing stable CRC cells were subsequently infected with lentivirus containing single guide RNA targeting NSD2 (targeted DNA sequence, 5′-CTTACTTCCCGGGTGTTTAA-3′, PAM sequence TGG) and selected with neomycin. Cells were then distributed into a 96-well plates and cultured for 48 h. Afterwards, NSD2-knockout (KO) screening was performed and single stable NSD2-KO CRC cells were established.

### Ectopic NSD2 overexpression

The full-length NSD2 cDNA or the catalytic inactive NSD2 (Y1179A, NSD2-Mut, the sequence was listed [11]) was inserted into the lentiviral GV369 vector (Genechem). The construct, along with the lentivirus package plasmids (Helper 1.0 and Helper 2.0), were co-transfected to HEK-293 cells. Lentiviral particles (MOI 10) were added to CRC cells (cultured in polybrene-containing complete medium, at 60% confluence). The stable cells were selected by puromycin (2.5 μg/mL) for five passages. NSD2 expression in stable cells was verified by the qRT-PCR and western blotting assays. Control cells were transduced with the empty vector.

### Cell viability analyses

CRC cells with applied genetic modifications were seeded in 96-well tissue culture plates at 4000 cells per well. Cells were cultured for 96 h and Cell Counting Kit-8 (CCK-8) optical density (OD) absorbance was tested at 490 nm in each well.

### Transwell assays

Transwell chambers (8 μm pore size) were purchased from BD Biosciences (Shanghai, China). CRC cells with applied genetic modifications were added to a transwell chamber upper compartment at 5 × 10^4^ cells per chamber (in basic medium). The lower compartment was filled with complete medium with 10% FBS. Cells were allowed to migrate for 24 h at 37 °C. Afterwards, cells from the upper compartment were removed very carefully by using a cotton swab. Cells that migrated to the lower face were fixed, stained, and counted manually. For invasion assays, Matrigel (Sigma) was coated on the lower side of the transwell chamber.

### Colony formation assay

CRC cells with applied genetic modifications were seeded into 10 cm dishes at 4 × 10^3^ cells/dish and cultured in complete medium. The medium was renewed every 48 h and cells were cultured for a total of 10 days. Afterwards, cells were washed and stained. The number of big colonies (with >50 cells in each colony) was manually counted.

### EdU staining

CRC cells with applied genetic modifications were seeded into a 96-well plate at a density of 4 × 10^3^ cells/well. Cells were cultured for additional 96 h. Next, an 5-ethynyl-20-deoxyuridine (EdU) Apollo-567 Kit (RiboBio, Guangzhou, China) was employed to quantify cell proliferation. EdU and 4′,6-diamidino-2-phenylindole (DAPI) dyes were both added to CRC cells and visualized under a fluorescent microscope (Leica, Shanghai, China). The EdU-positive nuclei ratio (% vs. DAPI) was always recorded.

### TUNEL staining

CRC cells with applied genetic modifications were seeded into a 96-well plate at a density of 4 × 10^3^ cells/well. Cells were cultured for additional 96 h. Detailed protocols of terminal deoxynucleotidyl transferase dUTP nick end labeling (TUNEL) staining and data quantification were described in our previous study [30]. The TUNEL-positive nuclei ratio (% vs. DAPI) was recorded.

### Other apoptosis-related assays and cell cycle analyses

Other apoptosis-related assays, including caspase-3 and caspase-9 activity assays, single-strand DNA (ssDNA) enzyme-linked immunosorbent assay, and mitochondrial depolarization detection by JC-1 staining were described in detail in our previous studies [7, 8, 31, 32]. Cell cycle distribution assay by propidium iodide staining and fluorescence activated cell sorting (PI-FACS) was reported previously [31].

### Xenograft studies

From the Experimental Animal Center of Soochow University Medical School, nude mice (half female and half female, 5–6 week of age, weighted at 18.1–18.5 g) were obtained (Suzhou, China). All nude mice were maintained under standard conditions, with 12 h dark/12 h light cycle, 24 ± 2 °C temperatures, and 50 ± 10% humidity, and free access of water and food. CRC cells with applied genetic modifications (6 × 10^6^ cells per mouse, in 200 µL of Matrigel-DMEM solution, FBS-free) were subcutaneously (s.c.) injected to the flanks of nude mice. Tumor volume was calculated using the descried formula [8]. All animal protocols were approved by Institutional Animal Care and Use Committees (IACUC) and Ethics Committee of Wenzhou Medical University.

### Statistics analyses

The quantitative data in this study were presented as mean ± SD. All statistics analyses were performed by the SPSS 23.0 statistical software (SPSS, Chicago, IL). One-way analysis of variance through the Scheffe’s *f*-test was applied to compare difference between multiple groups. For statistical comparison between two groups, the two-tailed unpaired *T*-test (Excel 2007) was applied. *P*-values < 0.05 were considered statistically significant.

## Results

### NSD2 overexpression in CRC tissues and cells

**Fig. 1:**
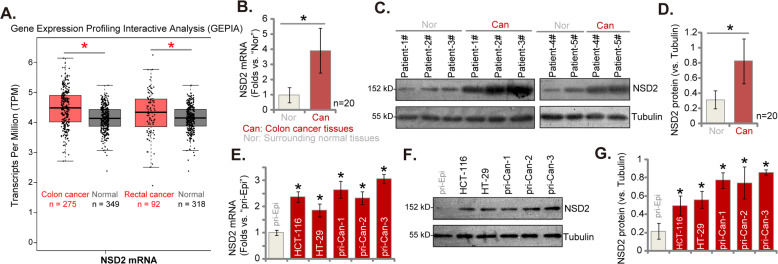
NSD2 overexpression in CRC tissues and cells. GEPIA database shows the relative *NSD2* mRNA expression in listed colon/rectal cancer tissues and normal tissues (**A**). *NSD2* mRNA and protein expression in colon cancer tissues (“Can”) and matched surrounding normal tissues (“Nor”) from 20 (*n* = 20) primary colon cancer patients were tested by qRT-PCR (**B**) and western blotting (**C**, **D**) assays, respectively. *NSD2* mRNA and protein expressions in listed CRC cells and primary colon epithelial cells (“pri-Epi”) were tested, with results quantified (**E**–**G**). Data were presented as mean ± SD. *P < 0.05 vs. normal tissues/“pri-Epi” cells.

Gene Expression Profiling Interactive Analysis database was first consulted to analyze the *NSD2* mRNA expression profile in CRC. As shown, *NSD2* mRNA levels in colon cancer tissues (*n* = 275) were significantly higher than those in normal colon tissues (*n* = 349) (Fig. 1A). In addition, *NSD2* mRNA upregulation was detected in the rectal cancer tissues (*n* = 92), whereas relative low expression was detected in normal rectal tissues (*n* = 318) (Fig. 1A). We next tested NSD2 expression in local CRC tissues. As described in our previous studies [7, 8], the colon cancer tissues (“Can”) and matched surrounding normal colon epithelial tissues (“Nor”) were from 20 primary colon cancer. qRT-PCR assay results (Fig. 1B) demonstrated that *NSD2* mRNA levels in colon cancer tissues were significantly higher than those in normal tissues. When testing NSD2 protein expression using western blotting assays, we found that NSD2 protein was overexpressed in colon cancer tissues in five representative patients (“Patient-1# to Patient-5#,” Fig. 1C). In addition, NSD2 blotting data of all 20 sets of tissues were quantified and combined. Results showed that NSD2 protein upregulation in colon cancer tissues was significant (*P* < 0.05 vs. normal tissues, Fig. 1D).

Next, a number of different human CRC cells, including the primary human colon cancer cells that were derived from three primary patients (“pri-Can-1/-2/-3” [7, 8]) and established CRC cell lines (HT-29 and HCT-116), were cultured. Expression of NSD2 was tested. Results showed that the *NSD2* mRNA expression in the primary and established CRC cells was significantly higher than that in the primary human colon epithelial cells (“pri-Epi” [7, 8]) (Fig. 1E). Furthermore, the NSD2 protein expression in CRC cells was also higher than that in pri-Epi cells (Fig. 1F, G). These results show that NSD2 is upregulated in CRC tissues and cells.

### NSD2 shRNA produces significant anti-cancer activity in CRC cells

**Fig. 2:**
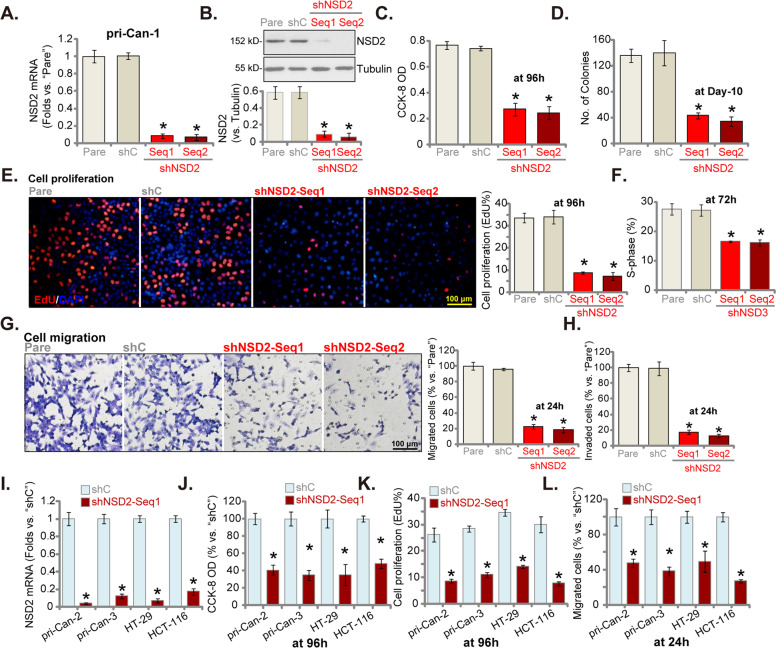
NSD2 shRNA produces significant anti-cancer activity in CRC cells. The primary human colon cancer cells (“pri-Can-1/-2/-3”, **A**–**L**) or established CRC cell lines (HT-29 and HCT-116, **I**–**L**) were transduced with applied lentiviral NSD2 shRNA (“shNSD2-Seq1/2”) or scramble non-sense control shRNA (“shC”), stable cells were established following selection by puromycin. Expression of *NSD2* mRNA (**A** and **I**) and listed proteins (**B**) were shown. Cells were further cultured for applied time periods, cell viability was tested by CCK-8 (**C**, **J**) and colony formation (**D**) assays. Cell proliferation was tested by nuclear EdU staining (**E**, **K**). Cell cycle distribution was tested by PI-FACS and S-phase cells were quantified (**F**). Cell migration and invasion were tested by “Transwell” (**G**, **L**) and “Matrigel Transwell” (**H**) assays, respectively. For all the in vitro functional assays, the exact same number of viable cells with different genetic modifications was initially plated into each well/dish (at “0 h” or “Day-0”). NSD2 protein expression was normalized to the loading control protein (**B**). “Pare” stands for the parental control cells. Data were presented as mean ± SD (*n* = 5). *P < 0.05 vs. “shC” cells. Experiments in this figure were repeated five times and similar results were obtained. Scale bar = 100 μm (**E**, **G**).

The two different lentiviral shRNAs targeting two non-overlapping sequences of NSD2 were generated and were named as shNSD2-Seq-1 and shNSD2-Seq2. The two were individually transduced to primary cultured human colon cancer cells, pri-Can-1 (see our previous study [7]). Stable cells were established after culturing cells in the puromycin-containing medium. The qRT-PCR assay results showed that *NSD2* mRNA levels decreased over 90% in NSD2 shRNA-expressing pri-Can-1 cells (Fig. 2A). Consequently, NSD2 protein expression was robustly decreased (Fig. 2B). To analyze the functional consequences of NSD2 silencing, we demonstrated that cell viability, or CCK-8 OD, was significantly decreased in NSD2 shRNA-expressing pri-Can-1 cells (Fig. 2C). Moreover, NSD2 shRNA significantly inhibited pri-Can-1 cell colony formation (Fig. 2D). In NSD2-silenced cells, the EdU-positive nuclei ratio was decreased, indicating proliferation inhibition (Fig. 2E). The cell cycle analyses demonstrated significantly decreased S-phase percentage in NSD2-silenced pri-Can-1 cells (Fig. 2F).

The “Transwell” assay and the “Matrigel Transwell” assay were employed to tested cell migration and invasion, respectively. Results showed that the numbers of migrated (Fig. 2G) and invaded (Fig. 2H) cells were largely decreased in pri-Can-1 cells expressing NSD2 shRNA. When compared to the parental control cells (“pare”), transfection of the lentiviral scramble control non-sense shRNA (“shC”) failed to significantly affect NSD2 expression (Fig. 2A, B) and pri-Can-1 cell functions (Fig. 2C–H).

We also studied whether NSD2 shRNA exerted a similar activity in other CRC cells. Primary human colon cancer cells derived from two other patients (pri-Can-2 and pri-Can-3, see our previous study [7]) as well as the established CRC cell lines (HT-29 and HCT-116) were infected with shNSD2-Seq-1 or shC. Stable cells were again selected by puromycin. When analyzing *NSD2* mRNA expression by qRT-PCR assays, we found that shNSD2-Seq-1 resulted in profound *NSD2* mRNA reduction in the CRC cells (Fig. 2I). Cell viability, or CCK-8 OD, was decreased in CRC cells after NSD2 silencing (Fig. 2J). Furthermore, NSD2 shRNA largely inhibited cell proliferation (EdU-positive nuclei ratio decrease, Fig. 2K) and decreased the migrated cell number (Fig. 2L) in the primary and established CRC cells. Therefore, NSD2 silencing by targeted shRNA induced viability reduction, proliferation inhibition, as well as migration and invasion inhibition in primary and established CRC cells.

### NSD2 shRNA induces apoptosis activation in CRC cells

**Fig. 3:**
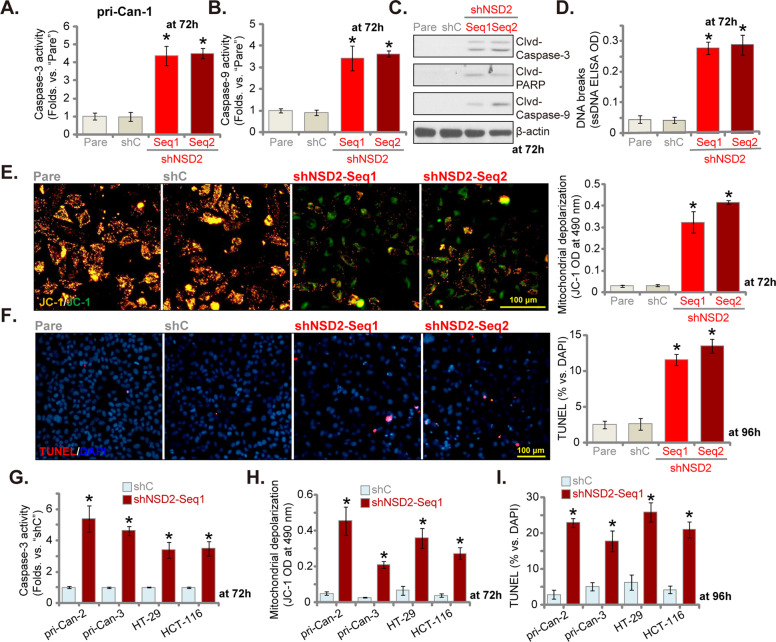
NSD2 shRNA induces apoptosis activation in CRC cells. The primary human colon cancer cells (“pri-Can-1/-2/-3”, **A**–**I**) or the established CRC cells (HT-29 and HCT-116, **G**–**I**) were transduced with applied lentiviral NSD2 shRNA (“shNSD2-Seq1/2”) or scramble non-sense control shRNA (“shC”), stable cells were established following selection by puromycin. Cells were further cultured for applied time periods, caspase-3/-9 activities (**A**, **B**, **G**), expression of apoptosis-associated proteins (**C**), and ssDNA contents (ELISA OD, **D**) were tested. Mitochondrial depolarization was examined via JC-1 dye assay (**E**, **H**). Cell apoptosis was tested by nuclear TUNEL staining (**F**, **I**) and results were quantified. “Pare” stands for the parental control cells. Data were presented as mean ± SD (*n* = 5). *P < 0.05 vs. “shC” cells. Experiments in this figure were repeated five times and similar results were obtained. Scale bar = 100 μm (**E**, **F**).

Activation of caspases is an initial step and characteristic marker of cell apoptosis [33–36]. As shown, the caspase-3 activity increased four- to fivefolds in stable pri-Can-1 cells expressing shNSD2 (Fig. 3A). The caspase-9 activity was increased as well (Fig. 3B). Moreover, western blotting assay results (Fig. 3C) demonstrated that caspase-3, caspase-9, and PARP were cleaved in NSD2-silenced pri-Can-1 cells. Indicating DNA damage, we found that ssDNA contents were significantly increased in pri-Can-1 cells following NSD2 knockdown (Fig. 3D). In addition, NSD2 shRNA induced significant mitochondrial depolarization, which was evidenced by accumulation of JC-1 green monomers (Fig. 3E). The above results indicated that NSD2 silencing by targeted shRNA induced activation of mitochondria-dependent intrinsic apoptosis cascade [34, 36, 37] in pri-Can-1 cells. To support apoptosis activation, we found that TUNEL-positive nuclei ratio was significantly increased in pri-Can-1 cells expressing NSD2 shRNAs (Fig. 3F). As expected, the scramble control non-sense shRNA (“shC”) failed to induce significant activation of caspases and apoptosis in pri-Can-1 cells (Fig. 3A–F).

In other primary cancer cells, pri-Can-2 and pri-Can-3, as well as in established CRC cell lines (HT-29 and HCT-116), stable NSD2 silencing by the shNSD2-Seq-1 (see Fig. 2) similarly increased caspase-3 activity (Fig. 3G) and induced mitochondrial depolarization (JC-1 green monomers accumulation, Fig. 3H). Significant apoptosis activation, reflected by increased TUNEL-positive nuclei ratio, was detected as well in NSD2-silenced CRC cells (Fig. 3I). Together, NSD2 silencing by targeted shRNA induced apoptosis activation in CRC cells.

### NSD2-KO inhibits CRC cell proliferation and migration, and inducing apoptosis activation

**Fig. 4:**
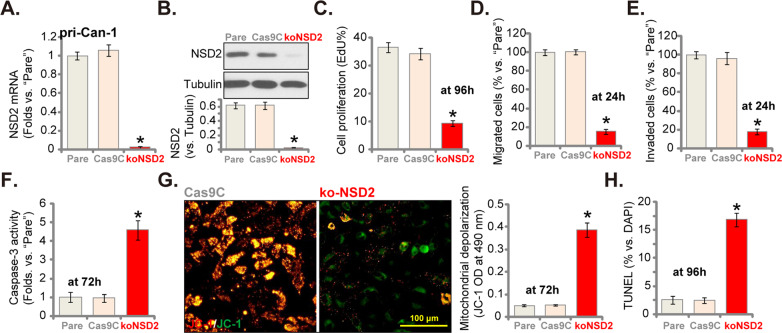
NSD2-KO inhibits CRC cell proliferation and migration, and inducing apoptosis activation. Stable primary human colon cancer cells, pri-Can-1, carrying a lentiviral CRISPR/Cas9-NSD2-knockout (KO) construct (“koNSD2”) or CRISPR/Cas9 control construct (“Cas9C”), were established. Expression of NSD2 *mRNA* (**A**) and listed proteins (**B**) were shown. Cells were further cultured for applied time periods, cell proliferation (by recording EdU-positive nuclei ratio, **C**), migration and invasion (“Transwell assays”, **D**, **E**), as well as the relative caspase-3 activity (**F**), mitochondrial depolarization (JC-1 dye assays, **G**), and cell apoptosis (by recording nuclear TUNEL ratio, **H**) were tested, with results quantified. “Pare” stands for the parental control cells. Data were presented as mean ± SD (*n* = 5). *P < 0.05 vs. “Cas9C” cells. Experiments in this figure were repeated five times and similar results were obtained. Scale bar = 100 μm (**G**).

To exclude the possible off-target effect of the applied NSD2 shRNAs and to further support the oncogenic role of NSD2 in CRC cells, the CRISPR/Cas9 gene-editing method was employed to completely knock out NSD2 in CRC cells. As described, a CRISPR/Cas9-NSD2-KO construct was transduced to pri-Can-1 cells. Single, stable cells were established via KO screening (see “Methods”) and these cells were named as “koNSD2” cells. As compared to cells expressing the CRISPR/Cas9 control construct (Cas9C), *NSD2* mRNA (Fig. 4A) and protein (Fig. 4B) expressions were almost completely depleted in the koNSD2 pri-Can-1 cells. CRISPR/Cas9-induced NSD2-KO in pri-Can-1 cells potently inhibited cell proliferation (EdU-positive nuclei ratio decrease, Fig. 4C) and decreased the number of migrated cells (Fig. 4D) and invaded cells (Fig. 4E). Moreover, in koNSD2 cells, the caspase-3 activity was significantly increased (Fig. 4F). Mitochondrial depolarization, tested by the JC-1 green monomer accumulation (Fig. 4G), was also detected. Significant apoptosis activation was observed as well in the NSD2-KO pri-Can-1 cells, as the TUNEL-positive nuclei ratio was significantly increased (Fig. 4H). Therefore, NSD2-KO inhibited cell proliferation–migration and induced apoptosis activation in CRC cells. Cas9C did not significantly affect NSD2 expression (Fig. 4A, B) and pri-Can-1 cell functions (Fig. 4C–H).

### Ectopic overexpression of NSD2 can expedite CRC cell proliferation and migration

**Fig. 5:**
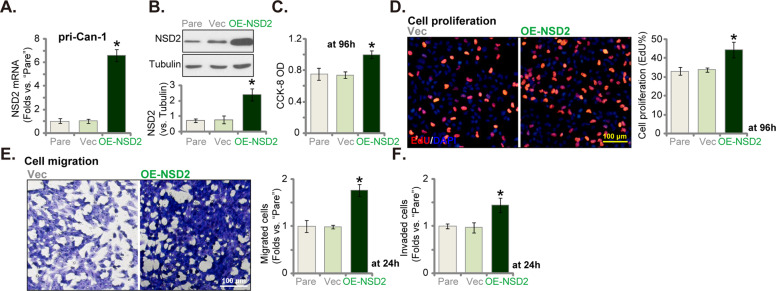
Ectopic overexpression of NSD2 can expedite CRC cell proliferation and migration. Stable primary human colon cancer cells, pri-Can-1, expressing a lentiviral construct encoding the full-length *NSD2* cDNA (“OE-NSD2”) or empty vector (“Vec”), were established. Expression of NSD2 *mRNA* (**A**) and listed proteins (**B**) were shown. Cells were further cultured for applied time periods; cell viability (CCK-8 OD, **C**), proliferation (EdU staining assays, **D**), migration, and invasion (“Transwell assays”, **E**, **F**) were tested, with results quantified. “Pare” stands for the parental control cells. Data were presented as mean ± SD (*n* = 5). *P < 0.05 vs. “Vec” cells. Experiments in this figure were repeated five times and similar results were obtained. Scale bar = 100 μm (**D**, **E**).

We have shown that shRNA-induced silencing or CRISPR/Cas9-induced KO of NSD2 potently inhibited cell proliferation and induced apoptosis in CRC cells. It is hypothesized that ectopic overexpression of NSD2 might expedite CRC cell proliferation and migration. Therefore, a lentiviral construct encoding the full-length *NSD2* cDNA was synthesized and transduced to pri-Can-1 cells. Stable cells were established via selection using puromycin-containing medium. These cells were named as “OE-NSD2” cells. As shown, *NSD2* mRNA levels in the OE-NSD2 cells increased over six folds (vs. control cells with empty vector, Fig. 5A). NSD2 protein expression was significantly elevated as well (Fig. 5B).

CCK-8 assay results confirmed that pri-Can-1 cell viability OD was increased after NSD2 overexpression (Fig. 5C). Furthermore, the EdU-positive nuclei ratio was significantly increased in OE-NSD2 cells (Fig. 5D), indicating that NSD2 overexpression expedited pri-Can-1 cell proliferation. In addition, as compared to vector control cells, pri-Can-1 cell migration (Fig. 5E) and invasion (Fig. 5F) were accelerated in the NSD2-overexpressed pri-Can-1 cells. Together, ectopic NSD2 overexpression expedited CRC cell proliferation and migration. The empty vector (“Vector”) failed to alter NSD2 expression (Fig. 5A, B) and cell functions (Fig. 5C–F).

### H3K36 methylation, expression of multiple oncogenes, and Akt activation are decreased with NSD2 silencing or KO in CRC cells

**Fig. 6:**
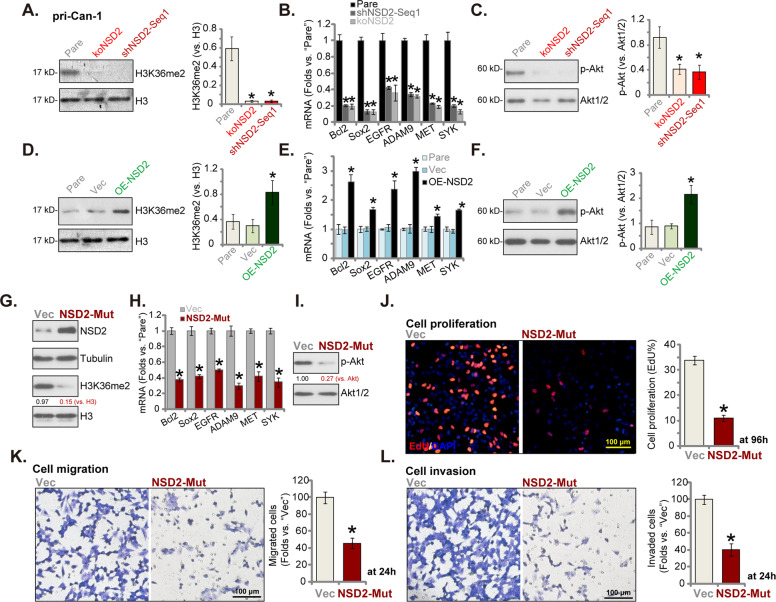
H3K36 methylation, expression of multiple oncogenes, and Akt activation are decreased with NSD2 silencing or KO in CRC cells. Stable pri-Can-1 cells, expressing shNSD2-Seq1, a lentiviral CRISPR/Cas9-NSD2-knockout (KO) construct, a lentiviral construct encoding the full-length NSD2 cDNA (“OE-NSD2”), or empty vector (“Vec”), were established and cultured. Expressions of listed proteins and mRNAs were tested by western blotting (**A**, **C**, **D**, **F**) and qRT-PCR assays (**B**, **E**), respectively. Stable pri-Can-1 cells with the lentiviral construct encoding the catalytic inactive NSD2 (Y1179A, “NSD2-Mut”) or empty vector (“Vec”) were established. Expression of listed proteins (**G**, **I**) and mRNAs (**H**) were shown. Cells were further cultured for applied time periods, cell proliferation (EdU staining assays, **J**), migration, and invasion (“Transwell assays”, **K**, **L**) were tested, with results quantified. “Pare” stands for the parental control cells. Data were presented as mean ±SD (*n* = 5). *P < 0.05 vs. “Pare”/“Vec” cells. Experiments in this figure were repeated five times and similar results were obtained.

It has been shown that the principal chromatin-regulatory activity of NSD2 is to catalyze dimethylation of H3K36me2, which is sufficient and necessary for transcription activation of multiple genes [11]. Several studies have performed RNA-sequencing studies to test differentially regulated mRNAs in cells with different NSD2 expression and have identified several important NSD2-dependent oncogenic genes [15, 16, 38]. The representative genes were tested here, including *ADAM9*, *EGFR* [15], *Sox2*, *Bcl-2* [16], *SYK*, and *MET* [38]. As shown, levels of H3K36me2 (Fig. 6A) and expressions of the oncogenic genes (Fig. 6B) were significantly downregulated in pri-Can-1 cells with shNSD2-Seq-1 (see Figs. 2 and 3) and CRISPR/Cas9-NSD2-KO construct (see Fig. 4). Existing studies have also proposed that NSD2 is important for Akt activation in different cancer cells [13, 15, 16, 25]. We here found that phosphorylated Akt was dramatically decreased in NSD2-silenced or NSD2-KO pri-Can-1 cells (Fig. 6C). On the contrary, in NSD2-overexpressed pri-Can-1 cells (Fig. 5), levels of H3K36me2 (Fig. 6D), expression of oncogenic genes (*ADAM9*, *EGFR*, *Sox2*, *Bcl-2*, *SYK*, and *MET*) (Fig. 6E), and Akt phosphorylation (Fig. 6F) were significantly increased (*P* < 0.05 vs. vector control cells).

Next, a lentiviral construct encoding the catalytic inactive NSD2 (Y1179A, NSD2-Mut [11]) was transduced to the primary pri-Can-1 cells and stable cells established after selection. The NSD2-Mut potently inhibited H3K36me2 in pri-Can-1 cells (Fig. 6G). NSD2-dependent genes (*ADAM9*, *EGFR*, *Sox2*, *Bcl-2*, *SYK*, and *MET*) (Fig. 6H) as well as Akt phosphorylation (Fig. 6I) were inhibited by the catalytic inactive NSD2. Functional studies showed that the mutant NSD2 potently inhibited pri-Can-1 cell proliferation (EdU-positive nuclei ratio reduction, Fig. 6I), migration, and invasion (Fig. 6J, K). These results further supported the oncogenic role of NSD2 in CRC cells.

### NSD2 shRNA inhibits pri-Can-1 xenograft growth in nude mice

**Fig. 7:**
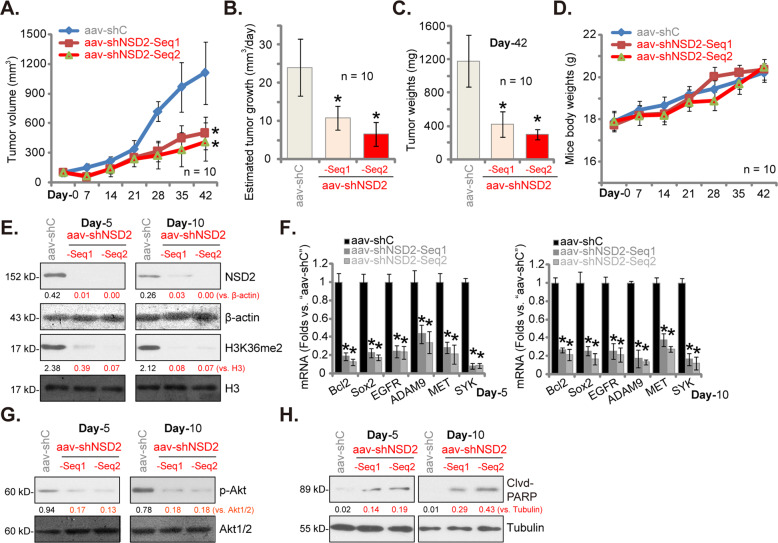
NSD2 shRNA inhibits pri-Can-1 xenograft growth in nude mice. The pri-Can-1 xenograft-bearing nude mice were subjected to intratumoral injection of AAV-packed shNSD2-Seq1 (“aav-shNSD2-Seq1”), AAV-packed shNSD2-Seq2 (“aav-shNSD2-Seq2”), or AAV-packed shC (“aav-shC”), daily for 10 consecutive days. Tumor volumes (**A**) and mice body weights (**D**) were recorded every week for 6 weeks. The estimated daily tumor growth, in mm^3^ per day, was calculated by the described formula (**B**). At Day-42, pri-Can-1 xenografts were carefully isolated and weighted individually (**C**). At Day-5 and Day-10, one tumor of each group was isolated and total six tumors were analyzed. Expressions of listed proteins and mRNAs were tested by western blotting (**E**, **G**, **H**) and qRT-PCR assays (**F**), respectively. Protein expression was quantified and normalized to the loading control (**E**, **G**, **H**). Data were presented as mean ± SD. Ten mice were in each group (*n* = 10) (**A**–**D**). *P < 0.05 vs. “aav-shC” groups.

At last, we studied the potential effect of NSD2 on CRC cell growth in vivo. As described, pri-Can-1 cells (6 × 10 ^6^ cells per mouse) were s.c. injected to the flanks of nude mice. Within 3 weeks, the pri-Can-1 xenografts were established and the volume of each single tumor was close to 100 mm^3^ (labeled as “Day-0”). The xenograft-bearing nude mice were randomly assigned into three groups, subjected to intratumoral injection of AAV-packed shNSD2-Seq1, AAV-packed shNSD2-Seq2, or AAV-packed shC. AAV injection was performed daily for 12 consecutive days. Tumor growth curve results (Fig. 7A) demonstrated that the pri-Can-1 xenograft growth was largely inhibited after shNSD2 AAV injection. The estimated daily tumor growth (in mm^3^ per day) was calculated by the following formula: [Tumor volume (in mm^3^) at Day-42 − tumor volume at Day-0]/42. The shNSD2 AAV-injected xenografts presented with significantly decreased daily tumor growth (Fig. 7B). At the end of the experiments (Day-42), all tumors in the three groups were isolated and weighted individually. Results showed that shNSD2 AAV-injected tumors were significantly lighter than shC AAV-injected tumors (Fig. 7C). The mice body weights, shown in Fig. 7D, were not significantly different between the three groups.

To examine signaling changes in vivo, at Day-5 and Day-10, one xenograft of each group was isolated and total six tumors were analyzed. Western blotting assays of tumor lysates found that levels of NSD2 protein and H3K36me2 levels were depleted in shNSD2 AAV-injected tumors (Fig. 7E). In addition, qRT-PCR assay results in Fig. 7F confirmed that mRNA expression of NSD2-associated oncogenes, including *ADAM9*, *EGFR*, *Sox2*, *Bcl-2*, *SYK*, and *MET*, was significantly decreased in shNSD2-injected pri-Can-1 xenografts. Akt activation, or phosphorylated Akt, was inhibited as well (Fig. 7G). On the other hand, levels of cleaved-PARP were increased in shNSD2 AAV-injected pri-Can-1 xenografts, indicating apoptosis activation (Fig. 7H). Therefore, in pri-Can-1 xenografts, NSD2 silencing by AAV shRNA injection decreased H3K36m2, expression of multiple oncogenes, and Akt activation.

## Discussion

Dysregulation of histone methylation will cause aberrant transcription activation and expression of cancer-related genes, participating in different pathological processes of cancer, including proliferation, metabolic reprogramming, EMT, and metastasis [9]. Targeting histone methylation regulators (lysine methyltransferases and lysine demethylases) has become a promising strategy for the management of CRC [9]. NSD2 specifically catalyzes H3K36me2, which will lead to an open conformation of chromatin to promote gene transcription [14]. Studies have revealed that NSD2 is important for the proliferation and/or survival of different types of human cancer cells, including myeloma cells, leukemia cells, prostate cancer cells, and osteoblastoma cells [11, 17, 21, 22, 39].

Our results implied that NSD2 could be an important oncogenic gene for CRC. *NSD2* mRNA and protein expression is significantly elevated in human colon cancer tissues, whereas relatively low NSD2 expression is detected in match surrounding normal tissues. NSD2 overexpression was also detected in established and primary human CRC cells. Importantly, NSD2 knockdown (by targeted shRNA) or KO (using CRISPR/Cas9 gene-editing method) potently suppressed CRC cell proliferation, cell cycle progression, migration, and invasion. Furthermore, significant apoptosis activation was detected in NSD2-silenced or NSD2-KO CRC cells. Conversely, ectopic overexpression of NSD2 expedited proliferation and migration of colon cancer cells. In vivo, the growth of the pri-Can-1 xenografts in nude mice was significantly inhibited by intratumoral injection of AAV-packed NSD2 shRNA. Therefore, NSD2 silencing can robustly inhibit CRC cell growth in vitro and in vivo.

Studies have shown that increased H3K36me2 in NSD2-overexpressed multiple myeloma cells is important for the transcription activation of several key oncogenes, including *SYK* and *MYK* [38]. He et al. [16] showed that NSD2-catalyzed H3K36me2 was required for the transcription activation of Bcl2 and Sox2 oncogenes in osteosarcoma cells. Kuo et al. [11] reported that NSD2-mediated H3K36me2 promoted expression of several oncogenic genes (*TGFA*, *PAK1*, *MET*, *RRAS2*, and many others) and initiate oncogenic programming [11]. Wang et al. [15] found that transcription activation of ADAM9-EGFR required NSD2 in triple-negative breast cancer cells.

Here we found that levels of H3K36me2 and expressions of NSD2-associated oncogenic genes, *ADAM9*, *EGFR*, *Sox2*, *Bcl-2*, *SYK*, and *MET*, were largely inhibited after NSD2 silencing or KO in CRC cells. Their levels were however increased with ectopic NSD2 overexpression. Furthermore, H3K36me2 and expressions of the oncogenic genes were decreased in NSD2 shRNA AAV-injected xenograft tissues. These results implied that NSD2-mediated H3K36me2 should be important for transcription activation and expression of multiple oncogenes in CRC.

Due to mutations in different genes (*PTEN* and *PI3KCA*, etc.) or constitutive activation of different receptor tyrosine kinases (RTKs) (i.e., epidermal growth factor receptor (EGFR), platelet-derived growth factor receptor, and fibroblast growth factor receptor), persistent activation of PI3K-Akt signaling is often detected in CRC [40–43], which is essential for cancer cell growth, proliferation, metastases, as well as metabolism reprogramming and apoptosis/therapy resistances [44]. Recent studies have shown that NSD2 is important for Akt activation. For example, Li et al. [20] found that NSD2 transcriptionally increased expression of Rictor, a key component of mammalian target of rapamycin complex 2, to promote Akt Ser-473 phosphorylation and activation. Wang et al. [15] reported that NSD2 increased transcription activation of ADAM9-EFGR to promote downstream Akt activation in triple-negative breast cancer cells. In ccRCC cells and osteosarcoma cells, NSD2 silencing also decreased Akt phosphorylation [13, 16]. Similarly, Yin et al. [45] reported that NSD2 overexpression promoted Akt activation in cervical cancer cells.

In the present study, we found that Akt activation was largely inhibited in primary CRC cells after NSD2 silencing or KO. On the contrary, ectopic overexpression of NSD2 increased Akt activation. In vivo, Akt activation was inhibited in AAV-NSD2 shRNA-injected pri-Can-1 xenografts. These results indicated that NSD2-driven CRC cell progression could be partially due to its role on regulating Akt activation. The underlying signaling mechanisms may warrant further characterizations.

## Conclusion

CRC ranks fourth in morbidity and second in mortality among all malignancies [1, 2]. It is still a deadly disease for many affected patients [1, 2]. In-depth understanding of the pathological mechanisms of CRC progression is vital for both early diagnosis and treatment for this devastating disease. The results of the current study suggest that NSD2 functions as a novel and vital oncogenic gene required for CRC growth in vitro and in vivo, and targeting NSD2 could be an important therapeutic strategy for CRC.

## Data Availability

All data are available upon request.
